# Unraveling the Link between Interferon-α and Systemic Lupus Erythematosus: From the Molecular Mechanisms to Target Therapies

**DOI:** 10.3390/ijms232415998

**Published:** 2022-12-15

**Authors:** Barbara Infante, Silvia Mercuri, Andrea Dello Strologo, Rossana Franzin, Valeria Catalano, Dario Troise, Emanuela Cataldo, Paola Pontrelli, Carlo Alfieri, Valentina Binda, Giulia Frontini, Giuseppe Stefano Netti, Elena Ranieri, Loreto Gesualdo, Giuseppe Castellano, Giovanni Stallone

**Affiliations:** 1 Unit of Nephology, Dialysis and Transplantation, Advanced Research Center on Kidney Aging (A.R.K.A.), Department of Medical and Surgical Sciences, University of Foggia, 71122 Foggia, Italy; 2 Nephrology, Dialysis and Transplantation Unit, Department of Emergency and Organ Transplantation, University of Bari Aldo Moro, 70124 Bari, Italy; 3 Unit of Clinical Pathology, Center for Molecular Medicine, Advanced Research Center on Kidney Aging (A.R.K.A.), Department of Medical and Surgical Science, University of Foggia, 71122 Foggia, Italy; 4 Department of Nephrology, Dialysis and Renal Transplantation, Fondazione IRCCS Ca’ Granda Ospedale Policlinico, 20122 Milan, Italy; 5 Department of Clinical Sciences and Community Health, University of Milan, 20122 Milan, Italy

**Keywords:** systemic lupus erytrematosus, type-I interferons, interferon-α, lupus nephropathy

## Abstract

Systemic lupus erythematosus (SLE) is a chronic, systemic autoimmune disease with a wide range of clinical expressions. The kidney is often affected, usually within 5 years of the onset of SLE, and lupus nephropathy (LN) carries a high risk for increased morbidity. The clinical heterogeneity of the disease is accompanied by complex disturbances affecting the immune system with inflammation and tissue damage due to loss of tolerance to nuclear antigens and the deposition of immune complexes in tissues. Several studies have reported that in human SLE, there is an important role of the Type-I-interferons (INF) system suggested by the upregulation of INF-inducible genes observed in serial gene expression microarray studies. This review aims to describe the transduction pathways of Type-I-interferons, in particular INFα, and its immune-regulatory function in the pathogenesis of SLE and, in particular, in LN. In addition, recent novelties concerning biologic therapy in LN will be discussed.

## 1. Introduction

**Figure 1:**
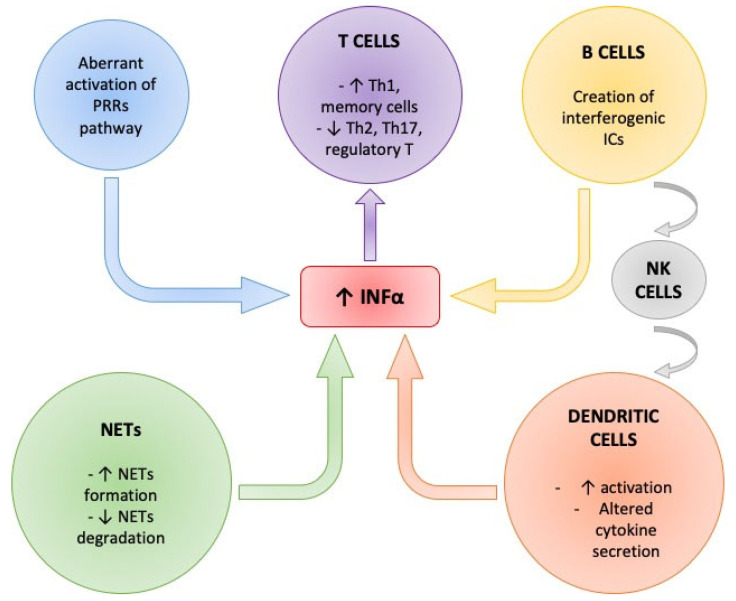
Summary of the main regulators of INFα in SLE.

Systemic lupus erythematosus (SLE) is systemic autoimmune disease with a wide range of clinical expressions [1]. The estimated incidence and prevalence of SLE vary considerably between geographic regions [2,3]. SLE is not related to a single cause but a complex interplay of environmental, genetic and epigenetic factors [4,5]. The clinical heterogeneity of the disease is accompanied by complex disturbances affecting the immune system with inflammation and tissue damage [6]. The kidney is often affected, usually within 5 years of the onset of SLE, and lupus nephritis (LN) carries a high risk for increased morbidity [7,8]. In the presence of SLE, autoreactive, long-lived plasma cells and memory T-cells memorize their immunization against autoantigens. The hyperactivation of antigen presenting cells (APCs) results in the incorrect recognition of autoantigens and to an activation of mature autoreactive B-cells [9]. Moreover, there is an important role of the Type-I-interferons (INF) system [10,11]. It has been reported that increased levels of type-I-interferons are present in SLE and correlated to disease activity [12]. Further studies have demonstrated that type-I-interferons, and particularly Interferon-alpha (INFα), have a central role in SLE pathogenesis [13,14]. Moreover, there is evidence that INFα can activate transduction pathways that lead to an expression of INF-induced genes with immunoregulatory functions on immune cells raising the possibility that this cytokine may drive the autoimmune response in SLE [15]. Our review will focus on transduction pathways of Type-I-interferons, in particular INFα, and its immune-regulatory function in the pathogenesis of SLE and in particular in LN (Figure 1).

## 2. The Type-I-Interferons

Interferons (INFs) are a family of immune system mediators that play a key role in innate defense against pathogens [16]. IFNs’ production is induced by the recognition of pathogen-associated molecular patterns (PAMPs) from pattern recognition receptors (PRRs) of innate immune system. PRRs include Toll-like receptors (TLRs), particularly the TLR3, which binds dsRNA; TLR7 which, fixes ssRNA; and TLR8/TLR9, which binds dsDNA [17], retinoic acid-inducible gene-1(RIG-1)-like receptors (RLRs) and nucleotide oligomerization domain(NOD)-like receptors(NLRs). PRRs trigger the intracellular signaling cascades and lead to expression of inflammatory mediators that coordinate the elimination of infected cells. An aberrant activation of this pathway has been shown to trigger autoimmunity processes [18].

Type-I IFNs, the most important IFN type, comprise five classes in humans, namely, INFα, INFβ, INFγ, INFε and INFκ. In particular, INFα can be further divided into 12 subtypes [19], encoding by genes belonging to a family of 13 intronless genes on the short arm of chromosome 9 and exhibit homology in their primary, secondary and tertiary structures. They all exert functions via ligating to a shared ubiquitously expressed heterodimeric receptors (IFNAR) which is composed of IFNAR1 and IFNAR2 subunits. IFNAR2 subunit binds Type-I-interferons with relatively high affinity, whereas the IFNAR1 subunit has no detectable affinity for them but is absolutely necessary for signal transduction from the receptor complex and the biological activity of Type-I-interferons. These activities are mediated to the receptor-associated protein tyrosine kinases janus kinase 1 (JAK1) and tyrosine kinase 2 (TYK2), which induced a formation of a transcription factor complex consisting of a signal transducer and activator of transcription 1 (STAT1) and STAT2. STAT1 and STAT2, after their phosphorylation, dimerize and translocate to the nucleus where they assemble with interferon regulatory factor-9 (IRF-9), constituting, finally, a factor complex named interferon-stimulated gene factor 3 (ISGF3). ISGF3 binds sequences of DNA named interferon-stimulated response elements (ISREs) in the promoters of interferon-regulated genes (IRGs) and induced a cellular antiviral state and a production of antiviral effectors [20]. In addition, STAT1 can be activated in its homodimers form by phosphorylation via Type-I-interferons receptors and bind to gamma activated sequences (GASs), which induces the activation of pro-inflammatory genes [21]. Moreover, the Interferon-JAK/STAT signal transduction pathway communicates with other signal transduction pathways, such as the innate pattern recognition receptors (PRRs), and induces expression of interferons genes. Another source of Type-I-interferons production are the neutrophil extracellular traps (NETs) produced by a specific neutrophilic cell deaths pathway termed “NETosis”, where neutrophils extrude nuclear material such as histones, granular, decondensed chromatin and cytoplasmic proteins in a web-like form that wraps pathogens. SLE patients have an increased NET formation because lupus neutrophils can be primed by Type-I-interferons after the exposure to SLE-derived anti-ribonucleoprotein antibodies, they also have an impaired capacity to degrade NETs which expose nucleic acids to autoantibodies and contribute to production of interferogenic immune complexes [22,23].

## 3. Role of Interferon-Alpha Production in SLE

INFα regulates autoimmune responses and carries out its activity on multiple target T-cells and pathways and modulates mechanisms acting on maturation of myeloid DC, promoting development of CD4+ T-cells and the differentiation of B-cells, priming monocytes and neutrophils in SLE [24]. Increased levels of INFα could be linked with the disease activity and disease flares [25]. An accurate method to measure INF signatures has not been established; however, recently galectin-9 (Gal-9) was proposed as a biomarker for IFN-gene signatures [26].

Thus, the INFα production is regulated in a complex manner and a large number of cells are involved in this process.

## 4. Interferon-Alpha and Dendritic Cells (DCs)

Dendritic cells (DCs) are important in immune responses and in the maintenance of self-tolerance. They are considered a bridge between innate and adaptive immune response, playing a major role in initiation, amplification and perpetuation of the SLE disease.

**Figure 2:**
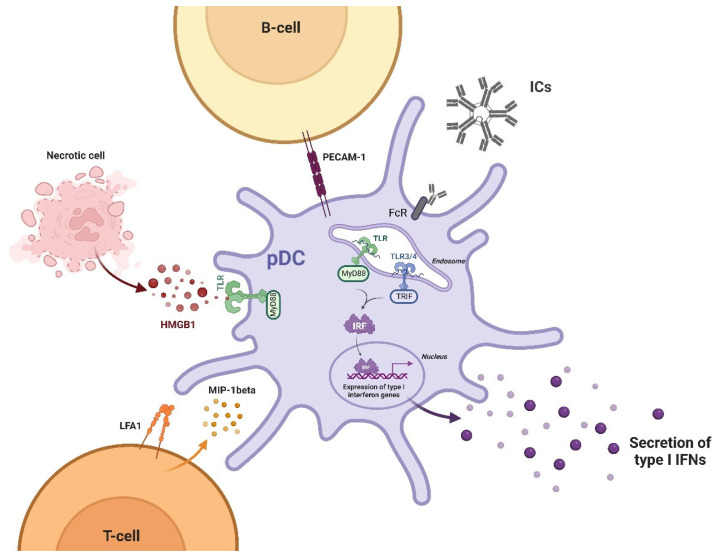
Regulation of INFα production by plasmacytoid DCs.

DC arise from the pluripotent bone marrow CD34+ hematopoietic progenitor cells (HPCs) and, based on their phenotypic and functional characteristics, can be divided into two classes: myeloid DCs (cDCs) and plasmacytoid DCs (pDCs). pDCs are considered the main source of INFα expressing TLR7 and TLR9 in their endosomal membranes. Their location in steady state is within the blood and secondary lymphoid organs and they can migrate into inflamed tissues such as skin and kidney once activated [27], promoting the interferons production [28]. pDCs can release INFα after the exposition to several stimuli (Figure 2).

The INFα production is regulated in a complex manner. pDCs are considered the main source of INFα and release INFα after the exposition to several stimuli. Immunecomplexes (ICs) bind immunoglobulin gamma Fc receptor IIa (FcgammaRIIa) on the surface of pDCs and interact with TLRs inducing INFα production by two different pathways: the MyD88 one and the TRIF one. The MyD88 pathway is activated by all TLRs, except TLR3, while the TRIF pathway is activated only by TLR3 and TLR4. This leads to the activation of the interferon regulatory factor (IRFs) pathway, resulting in INFα transcription. Peripheral B-cells enhance IFNα production by pDCS by cell-to-cell contact and this mechanism is dependent on the platelet endothelial cellular adhesion molecule (PECAM-1). Another source of INFα production from pDCs is a ubiquitous nuclear protein named high mobility group box-1 protein (HMGB1) released by necrotic cells that can trigger inflammation binding TLRs. T-cells are extremely potent in promoting the IFNα production by pDCs through the involvement of macrophage inflammatory protein-1 beta (MIP-1Beta), and the integrin lymphocyte function-associated antigen-1 (LFA-1).

Immunecomplexes (ICs) found in SLE patient’s sera are endocytosed after binding to the low-affinity immunoglobulin gamma Fc receptor IIa (Fc gamma RIIa) on the surface of pDCs and transported into the endosomes where the nucleic acids content interacts with TLRs inducing INFα production by two different pathways: the MyD88 one and the TRIF one. The MyD88 pathway is activated by all TLRs, except TLR3, and recruits effectors molecules such as members of the interleukin-1 receptor associated kinase (IRAK) family. This activates tumor necrosis factor receptor-associated factor 6 (TRAF6) and permits the transduction signal pathways such as the NF-kB one, the mitogen-activated protein kinases (MAPKs) one and the interferon regulatory factor (IRFs) ones. The TRIF pathway is activated only by TLR3 and TLR4, and has the ability to activate NF-kB and MAPKs promoter and the interferon regulatory factor pathways (IRFs) [29]. All of these molecular mechanisms result in transcription of various cytokines including INFα (Figure 2).

Another source of INFα production from pDCs is a ubiquitous nuclear protein named high mobility group box-1 protein (HMGB1) released by necrotic cells that can trigger inflammation binding TLRs and activate immune transduction pathways such as NF-kB pathway (Figure 2). High levels of HMGB1 were found in SLE sera and correlated to the levels of Type-I-interferons [30]. Elevated INFα is believed to induce a loss of tolerance and an enhancement of major histocompatibility complex (MHC) class-I and II on DC for antigen presentation and therefore an hyperactivation of immune responses and a persistence of autoimmune reactions [31].

Henault et al. reported that IgE antibodies specific for double-stranded DNA (dsDNA) also activated plasmacytoid DCs (pDCs) in SLE, leading to the secretion of substantial amounts of interferon-α (IFN-α) [32]. Interestingly, the concentration of dsDNA-specific IgE found in patient serum correlated with disease severity and with pDC function.

Although plasmacytoid DCs play a major role in SLE pathogenesis, myeloid DCs also play an important role displaying an altered pattern of cytokine secretion leading to a massive production of proinflammatory cytokines, such as IL6 and TNFα, and, therefore, an increased autoreactive T cell activation [33]. Circulating myeloid and plasmacytoid DCs are reduced in patients with active SLE compared to those in the remission state. Interestingly, both these subsets of immature DCs infiltrate the kidney of SLE [34].

## 5. Interferon-Alpha and B-Cells in SLE

B-cells development is tightly controlled and the survival of the ones which react against self-antigens are prevented by central and peripheral tolerance mechanisms. In SLE, self-reactive naive B-cells are not removed, and they are detectable in SLE sera due to a failure in their negative selection [35]. B-cells in SLE physiopathology have the ability to produce autoantibodies targeting nuclear components including DNA, ribonucleoprotein (RNP) particles (anti-Ro, anti-La, anti -Sm), histones and non-histone chromatin proteins and the capability of presenting autoantigens to T-cells through their B cell receptor [36,37].

There is some evidence about defects in central tolerance mechanisms controlling early stages of B cell development in the bone marrow identifying an inadequate B-cell-receptor (BCR) signaling as a crucial step in the development of an autoimmune response [38,39].

Altered peripheral tolerance has also been identified showing an aberrant and raised recombination-activating gene (RAG) expression in peripheral B-cells resulting to a mutation of BCR and, thus, development of autoreactive B lymphocytes [40]. An important cytokine for the development and selections of B-cells is B-cell activating factor (BAFF), also known as B-Lymphocyte stimulator (BLyS). In SLE patients, both forms of BLyS, the membrane one (mBLyS) and soluble one (sBLyS), are increased and correlated with other upregulated soluble mediators in SLE, such as INFα [41,42,43,44].

Peripheral B-cells enhance IFNα production by pDCS by cell-to-cell contact; this mechanism is dependent on the platelet endothelial cellular adhesion molecule (PECAM-1), also known as cluster of differentiation 31 (CD31) [45] (Figure 2).

Consequently, B-cells have a dual role regarding the IFNα production: creating interferogenic ICs and enhancing pDCs function, which can result in the Interferon-alpha signature typically seen in SLE patients [46].

## 6. Interferon-Alpha and T-Cells in SLE

T-cells contribute to SLE pathogenesis in many ways. Follicular T-helper cells were found to be elevated in peripheral blood from lupus patients and their interaction with B-cells is crucial in helping to produce autoantibodies, this type of interaction occurs in germinal centers located within B-cell follicles of secondary lymphoid organs [47]. An increased number of Th-17 that promotes neutrophil recruitment and activation and mediates inflammation have also been observed in lupus and it is inversely correlated to IL-2 production. Low IL-2 levels represent another hallmark of SLE. Moreover, T-cells direct the inflammation by induction of specific cytokines [48]. In particular, Natural Killer (NK)-cells are extremely potent in promoting the IFNα production by pDCs after their stimulation by interferogenic ICs via FcgammaRIIIA (Figure 2). Since its engagement is known to induce a higher affinity conformational state of chemokine such as NK cell–cell derived macrophage inflammatory protein-1 beta (MIP-1Beta), and the integrin lymphocyte function-associated antigen-1 (LFA-1) dependent cell to cell interaction, it has been hypothesized that they could be involved in the stimulation of pDCs mediated by NK cells. It has been reported that in SLE-increased serum levels of MIP-1beta, as well as an upregulation of LFA-1, an increased expression of Type-I INF-regulated genes is present [49]. The stimulatory effect of the NK-cells on the production of INFα by pDCs induced by RNA-containing ICs are strongly inhibited by monocytes through the secretion of mediators such as prostanoids, ROS (ROS) and TNF-alpha which could suppress INFα production by direct inhibitory effects on stimulatory NK cells, but SLE patients’ monocytes have a less efficient negative regulation on INFα production. That observation makes them an important part of the feedback mechanism that limits the autoimmune reaction driven by INFα [50]. Moreover, INFα prolong survival and promote the Th1 profile differentiation of CD4 T-cells, the generation of memory cells and suppress the development of Th2, Th17 and regulatory T-cells [51].

## 7. Other Regulators of INFalpha Production

There are many other regulators of the INFα production and patients with SLE may have dysfunctions in different regulatory pathways. Negative regulators are: pDCs-specific antigen named blood dendritic cell antigen-2 (BDCA-2) that can suppress induction of INFα by a mechanism dependent on calcium mobilization and protein-tyrosine phosphorylation [52]. Immunoglobulin-like transcript-7 (ILT-7), that are selectively expressed in human pDCs, inhibits the transcription and secretion of Type-I interferon [53].

## 8. Environmental Factors and Interferon-Alpha Production

The Type-I INF system can be activated by a number of environmental factors that can induce an SLE syndrome or trigger a flare of the disease. Exposures to ultraviolet (UV) wavelengths of sunlight is the most well-known of them. In fact, it can damage genomic DNA and cause cell death with the release of potential autoantigens normally hidden, such as nucleoproteins, that can be recognized by autoantibodies forming interferogenic immune complexes that induce Type-I INF production by pDCs in the skin. In addition, the DNA released by cells can act as immune stimulants and activate an intracellular signal transduction cascade dependent on a stimulator of interferon genes (STING)-protein, leading to an homodimerization of IRF3 and a transcription of a variety of gene targets including Type-I-interferons [54]. Moreover, UV light can induce release of reactive oxygen species (ROS) causing pyrimidine dimer formation in DNA that eases interferogenic ICs formation [55]. Infections can also trigger a disease flare because microbial DNA or RNA can be recognized by receptors and induce Type-I-interferons production [1]. Furthermore, estrogens contribute to the sex-based differences in epidemiology of the disease, females displayed an up-regulated Type-I interferon genes signature in B-cells and the gender difference was related to 17β-estradiol. An amplification of the INFα signaling in B-cells was found due to estrogens by down-regulating the expression of microRNAs(miRNAs) such as let-7e-5p, miR-98-5p and miR-145a-5p which increase the IkB-kinase epsilon (IKKε) transcript level significantly and subsequently phosphorylation of STAT1 increasing the pool of STAT1 available to associate with STAT2 and IRF9 to assemble the factor complex ISGF3 [56].

## 9. Renal Complication of SLE: The Role of the Complement System and IFNα

Kidney damage is the most frequent complication in SLE patients, increasing the morbidity and mortality [57].

The pathogenesis of LN involves both humoral and cell mediated immunity. It occurs in 50% of SLE [56]. LN pathological manifestations involve glomeruli, tubules, interstitium and vessels.

Glomerular lesions depend on the site of deposition of the immune-complexes. The most recent histological classification identifies six immunohistological classes [58,59,60].

Humoral immunity is mainly responsible for glomerular damage and is mediated by different mechanisms, such as the deposition of circulating preformed immune-complexes (ANA, anti dsDNA or anti-C1q), the formation of in situ immunocomplexes cross-reacting with nuclear antigens or nucleosomes bound to resident renal cells. These mechanisms trigger a cytotoxic and complement mediated glomerular damage [61].

The loss of immune tolerance to nuclear autoantigens is a pivotal mechanism triggering the abnormal production of autoantibodies. Genetic mutations compromising apoptosis, complement mediated dead cells opsonization and phagocytosis cause an increased exposure of the immune system to nuclear antigens. These intracellular autoantigens are made immunogenic by caspases and then exposed on the cell surface, becoming ANA target [62].

In this scenario, the glomerular deposition of immune complexes is mainly caused by their massive production that over-rides clearance pathways; in addition, the involvement of an increasing number of polyclonal autoantibodies classes, such as IgG IgA and IgM, causes the so called “full house” pathognomonic histological pattern [63]. The massive glomerular deposition of immune complexes activates the classical complement system. Complement activation is a double-edged sword in the LN and can act as both friend and foe [64].

In physiological conditions, complement mediates the safe clearance of circulating immune complexes and cellular debris and facilitates B-cell tolerance to self-antigens. In SLE patients, a deficit in complement system provokes a reduction in immune complexes clearance. This scenario leads to an immune complex accumulation, mainly in glomerular vessels, which provokes the activation of the classic complement pathway, the deposition of C1q, C4b and C3b/iC3b, and the release of soluble anaphylatoxins, such as C3a, C4a and C5a. Their production triggers the leukocytes infiltration and consequently the release of pro-inflammatory mediators and tissue damaging factors. The damage of endothelial glomerular cells and glomerular basement membranes promoted also by the formation of the membrane attachment complex C5b-9 increases glomerular permeability. The complement system can be also activated on resident renal cells such as proximal tubular cells regardless of the deposition of immunocomplexes [63].

Moreover, the aberrant activation of the alternative complement pathway contributes to the release of soluble anaphylatoxins (C3a, C5a) and of C5b-9, which exacerbate the tubule interstitial inflammation and damage [65,66].

Therefore, the reduction in serum classical complement components, such as C1q, C2, C3 and C4 is associated with the occurrence, development and prognosis of LN [67].

The safe removal of immune complexes, of apoptotic cells and debris, is mainly mediated by C1q, which is downregulated in SLE patients.

It has been demonstrated that purified C1q and MBL facilitate apoptotic cells clearance. Indeed, apoptotic cells opsonization with C1q and MBL targeted these cells to DCs as well as to macrophages, increasing their clearance [68].

Interestingly, this finding is in line with the strong correlation between the inherited deficiency of early complement component with SLE. In particular, genetic deficiencies of the early components from the CP are associated with the development of SLE, mainly C1q, C1r/C1s, C4 and C2 [69]. The role of complement system in SLE pathogenesis has been demonstrated in mice models. Indeed, C1q and C4 deficient mice developed glomerulonephritis and a lupus-like syndrome. NZB/W and MRL/lpr are the two best studied models that spontaneously develop a lupus-like syndrome [70]. Interestingly, in the MRL/lpr mouse, the C1q deficiency, and not the C3 deficiency, accelerated the development of glomerulonephritis [71]. Conversely, the C5 blocking prevented the in the NZB/W model [72].

Besides systemic complement dysregulation and glomerular involvement, LN is also characterized by local synthesis of the complement component C1q. Indeed, in a mice model of progressive LN, myeloid DCs activate in situ complement system and produce complement molecules, such as C1q in tubulo-interstitium [73]. C1q contributes to tissue damage, not only by the opsonization and the clearance of dying cells, but also by the production of proinflammatory cytokines by DCs [68]. Moreover, C1q contributes to the humoral and cell-mediated immunity activation. Indeed, it induces self-antigen specific T-cells activation by DCs and their Th1 differentiation.

Since the aberrant role of downstream complement activation and the necessity to maintain the C1q clearance ability has been demonstrated, specific complement inhibitors have been developed. Interestingly, Tomlinson et al. used CR2, that has natural affinity for C3d, to tag apoptotic or damaged cells and selectively protect renal cells by complement attack [74]. More recently, a novel complement C3 inhibitor (CRIg/FH) has been evaluated in the treatment of LN in MRL/lpr lupus mice and demonstrated to preserve renal function and glomerular complement activation. The promising results of the anti-C5 therapy in lupus prone mice have led to start several clinical trials with eculizumab. Preliminary support for the use of eculizumab in selected cases of SLE with renal involvement has been shown, especially in the presence of TMA, or in patients with refractory LN [75].

Unfortunately, these studies performed mostly in vitro and in animal models did not find an effective clinical or routine translation. In this context, understanding the IFN signature holds a promising potential strategy.

A growing body of evidence has demonstrated the pivotal role of IFN-α in the pathogenesis of LN. Indeed, serum IFN-α levels and polymorphism of interferon regulatory factor 5 gene are strictly correlated to SLE clinical activity and the risk of development of LN [76,77,78].

In addition, the degree of glomerular IFN-α expression well correlate with the severity of renal involvement [79].

Several classes of immune cells are involved in IFN production and the subsequent amplification of immune response. Tubulointerstitial inflammation triggered by the binding of the nucleic acids to the macrophages and renal DC Toll Like Receptors (TLRs), results in the formation of a tubulointerstitial infiltrate of naive T lymphocytes followed by their differentiation into T helper cells Th1, Th2 and Th17. Th1 cells produce several pro-inflammatory cytokines, such as interferon-γ(IFN-γ). Moreover, mesangial cells can contribute to type 1-IFN production, after the stimulation of TLRs. Indeed, Yung et al. demonstrated that the deposition of immunocomplexes in the mesangium accelerates the progression of inflammation, fibrosis and kidney damage by the inflammatory cytochines production and mesangial cells-derived proteins deposition [80]. Finally, myeloid and plasmacytoid DC play a pivotal role in the production of several pro inflammatory and pro fibrotic mediators, such as Type-I-interferons, TNF-a, IL-6, IL-1b and TGFb [34]. Several studies demonstrated the role of TLR7 in the induction of IFN-α production by pDCs in patients with SLE. Indeed, pDCs’ stimulation with a TLR7 ligand increased IFN-α production, which well correlated with SLE clinical activity [81,82].

The importance of TLR signaling in the pathogenesis of LN has been demonstrated in several animal models [83,84].

Several studies found, in humans, an increased TLR9-expression in proximal tubular cells and interstitial tissues [85,86,87]. Therefore, TLRs have a pivotal role in the proinflammatory signaling which mediates kidney damage in SLE patients.

Several studies demonstrated the role of IFN-α in the pathogenesis of LN in murine models. Indeed, the exposure of NZB/W mice to IFN-α has been correlated to autoantibody production, onset of proteinuria, and glomerular IgG deposition [88,89]. Conversely, the IFN-α/β downregulation in NZB mice, which were silenced in IFN-α/βR genes, reduced splenomegaly, anti-dsDNA antibody serum levels and renal involvement [90].

Moreover, due to the worsening of renal involvement in murine models of LN after the stimulation with polycytidylic acid, a strong inducer of Type-I IFN is reported. This stimulation provoked an increase in autoantibodies titers and in serum Ig, a massive renal lymphocyte infiltrate and a more severe renal damage [83,91].

Conversely, the deletion of Interferon regulatory factor 5 (IRF-5) coding genes provoked a reduction in IFNα and autoantibodies production, with an important improvement in kidney inflammation and damage [92,93].

Finally, the injection of adeno-IFNα viral particles in murine models of (NZM2328) rapidly induced an immune complex-mediated glomerulonephritis [94].

The pathogenetic role of Type-I IFN described in mice models was confirmed in patients suffering from LES. Indeed, it has been demonstrated that Serum IFNα levels correlate strongly with the degree of kidney involvement and damage. Moreover, IFNα gene expression and the interferon-inducible gene expression signature at level of peripheral blood cells has been linked with the development of active LN in wide range of studies [11,95,96].

From a renal perspective, renal biopsies of SLE patients show increased expression of IFN-inducible genes and pDCs infiltration in glomeruli of patients with active disease [97,98].

Type-I IFN contributes to renal damage by enhancing the massive production of proinflammatory cytokines, and, therefore, increasing autoreactive T-cell activation. Moreover, INFα promotes the Th1 profile differentiation of CD4 T-cells, the generation of memory cells and suppressed the development of Th2, Th17 and regulatory T-cells. Finally, it may induce podocyte damage. Indeed, the exposure of human and murine podocytes with IFN-α increased the expression of autophagy markers (LC3B-II) and decreased p62 expression in a time and dose dependent way. Moreover, the stimulation of human and mice podocytes with IFN-α downregulated mTOR signaling and induced autophagy [99].

Regarding the effect of Type-I IFN on podocytes in glomerulonephritis, Migliorini et al. demonstrated that IFNs consistently activated human and mouse podocytes and parietal epithelial cells to express numerous IFN-stimulated genes. The effect of IFN-β was to significantly induce podocyte death and increase the permeability of podocyte monolayers, whereas IFN-α caused cell-cycle arrest and inhibited the migration of parietal epithelial cells. Both IFNs suppressed renal progenitor differentiation into mature podocytes [100].

Another piece of evidence confirmed an interferon response signature in infiltrating leukocytes in renal parenchima in LN, correlated with the same signature in peripheral blood cells. In renal biopsies from SLE patients, plasmacytoid DC and renal tubular cells were the major producer of IFN-alpha since they stained positive for MXA [101,102].

Finally, the study from Arazi et al., using a single-cell transcriptomics to study kidney samples obtained from patients with LN and living donor controls, revealed the complexity of immune populations in LN kidneys, identifying multiple disease-specific subsets of myeloid, NK, T and B-cells, and giving rise to several observations [103]. In particular, the authors calculated an interferon response score for each cell, defined as the average expression of several known interferon-stimulated genes (ISGs). Interestingly, the authors found a significant upregulation of this score in kidney cells compared with living donor controls.

A growing body of evidence demonstrated the involvement of NF-kB pathway in the proinflammatory signaling that mediate glomerular and tubulo-interstitial damage in LN patients. Indeed, NF-kB expression is increased in endothelial and mesangial cells in these patients [104]. Moreover, nuclear translocation of p50 and p65, two of the components of the canonical NF-kB pathway, was observed in renal tubular and interstitial cells [105]. The up-regulation of NF-kB correlates well with the impairment of renal function and the histological degree of kidney damage [106]. Type-I IFN, along with several proinflammatory cytokines, provokes a NF-kB hyper-activation, that amplifies cytokine burst and downregulates Klotho expression [107]. sKlotho is a soluble isoform of a transmembrane protein, produced by proximal and distal tubule renal cells. sKlotho has a pleiotropic anti-aging, anti-inflammatory, anti-fibrotic age and protective effect on several organs and tissues, such as kidney [108,109,110]. Therefore, the sKlotho down-regulation is associated with a worse renal outcome in patients with LN. Taken together, these observations suggest that IFN is important in both the inflammatory process and development of damage in LN.

## 10. Biologic Therapy in Lupus Nephritis

The traditional immunosuppressive drugs used for the treatment of LN include glucocorticoids or hydroxychloroquine associated to cyclophosphamide, Mycophenolate Mofetil, Azathioprine, Methotrexate and calcineurin-inhibitors [111]. Novel therapeutic agents have been developed and are currently under evaluation in several clinical trials, as summarized in Table 1. The most used at the present time are Belimumab and Rituximab [112].

Belimumab, an anti-B lymphocyte stimulator or Blys, has been the first approved biologic drug for non-renal SLE. A two-phase III trial, BLISS-52 and BLISS-76, reported a higher proportion of patients responsive to Belimumab than standard therapy. Belimumab offers additional benefit in patients with high disease activity [113,114,115].

Rituximab is a chimeric monoclonal antibody anti-CD20; randomized controlled trials failed to reveal its benefit in renal and non-renal SLE when combined with conventional immunosuppressive protocols [116,117]. However, efficacy of rituximab in adult and pediatric patients with refractory SLE manifestations, including renal disease was reported [118]. It is now recommended the use of rituximab in LN unresponsive to other induction treatments.

A phase-II study, CALIBRATE, aimed to investigate the sequential use of Rituximab and Belimumab in recurrent or refractory LN [119]. This study did not demonstrate an improvement in the complete or partial renal response by adding Belimumab or placebo to induction therapy consisting of steroids, cyclophosphamide and Rituximab. However, Kraaij et al. demonstrated that combining Rituximab and Belimumab effectively reduces ANAs and regressed excessive NET formation ex vivo while achieving significant clinical responses in patients with severe refractory SLE [120].

Obinutuzumab is a humanized monoclonal antibody IgG1 anti-CD20. It has lower complement-dependent cytotoxicity than Rituximab, but greater cellular cytotoxicity and antibody-dependent phagocytosis, and greater direct depleting effect on B-cells allowing rapid depletion of target lymphocytes with sustained efficacy [121]. In a phase II study (NOBILITY), improvements in lupus serological markers and in renal responses through week 104 were observed in patients with LN who received obinutuzumab plus standard therapies compared with standard therapies alone [122]. In January 2021, the FDA approved Voclosporin, a calcineurin inhibitor, for LN treatment in combination with mycophenolate after two pivotal trials, 48-week phase IIb RCT (Aurinia Urinary Protein Reduction in Active Lupus With Voclosporin [AURA-LV]) and a 52-week phase III RCT (AURORA 1). The use of this drug shows a reduction in proteinuria and preserved renal function [123].

In recent years, several therapeutic approaches targeting IFN-alpha have been studied: Sifalimumab, a fully human anti-IFNα monoclonal antibody and Rontalizumab, a recombinant humanized monoclonal anti-IFNα antibody. A phase IIb placebo-controlled study tested the efficacy and safety of sifalimumab in 431 patients with SLE [124]. The study met its primary endpoint (percentage of patients with SRI-4 response at week-52). Rontalizumab was tested in extrarenal SLE, but did not meet its primary endpoint in a phase II study.

Other studies provided evidence for the efficacy and safety of anifrolumab, a human monoclonal antibody to the Type-I IFN receptor subunit 1, which blocks the action of Type-I IFNs, for moderately to severely active SLE [125,126,127]. In July 2021, anifrolumab was approved in the USA for the treatment of moderate to severe SLE patients who are receiving standard therapy.

**Table 1:** Current biological therapy in Lupus Nephritis (LN).

Therapy	Mechanism of Action	Current Development Stage	Refs
Belimumab	Anti-B lymphocyte stimulator	Phase III	[113,114,115]
Rituximab	Chimeric monoclonal antibody anti-CD20	Phase II	[120]
Obinutuzumab	Humanized monoclonal antibody IgG1 anti-CD20	Phase II	[122]
Voclosporin	Calcineurin inhibitor	Phase II	[123]
Sifalimumab	Fully human monoclonal antibody anti-IFNα	Phase II	[124]
Anifrolumab	Human monoclonal antibody anti-Type-I IFN receptor subunit 1	Phase III	[125,126,127]

## 11. Conclusions

Type-I-interferons production is affected by interactions between plasmacytoid DC and many other cell types as well as environmental factors. Several signal transduction pathways and mechanisms are implicated, and it is likely that different patients may have dysfunctions in different regulatory pathways. Understanding the exact mechanisms behind the interferon signature and production is important because it can allow us to find new ways to downregulate the Type-I interferon system in SLE, and new molecules to target for therapeutic purposes, as well as define subgroups of patients that would benefit from a specific treatment. These novelties might be of particular interest in the early identification and targeted treatment of LN.

## Data Availability

Not applicable.
